# Plasma Micronutrient Profile of Prostate Cancer Cases Is Altered Relative to Healthy Controls—Results of a Pilot Study in South Australia

**DOI:** 10.3390/cancers15010077

**Published:** 2022-12-23

**Authors:** Varinderpal S. Dhillon, Permal Deo, Michael Fenech

**Affiliations:** 1 Health and Biomedical Innovation, Clinical and Health Sciences, University of South Australia, Adelaide 5000, Australia; 2 Genome Health Foundation, North Brighton, Adelaide 5048, Australia

**Keywords:** plasma concentration, prostate cancer (PC), micronutrients, elements

## Abstract

**Simple Summary:**

Prostate cancer remains one of the most common and fatal cancers in men. However, the nutrient deficiencies and excesses that are associated with it remain undetermined. We, therefore, investigated the concentration of micronutrients in the plasma of men diagnosed with late onset prostate cancer and compared their results to those for plasma collected from healthy age-matched controls. We found that the plasma concentrations of lutein, lycopene, α-carotene, β-carotene and selenium were significantly reduced, and the levels of iron, sulphur and calcium were significantly increased in prostate cancer cases relative to controls. Measurement of the concentration profile of these micronutrients may identify those men with a high risk of prostate cancer and inform the design of future dietary intervention studies that could reduce the risk of this insidious cancer.

**Abstract:**

Emerging evidence suggests possible roles of micronutrients in cancer prevention. The study was designed to test the hypothesis that the concentration profile of plasma micronutrients (i.e., the nutriome) in prostate cancer patients is different from that of healthy controls. Plasma samples from 116 Caucasian men diagnosed with late onset of prostate cancer and 132 matched controls from the South Australian population were collected and analysed for their concentration of micronutrients. Plasma concentrations of lutein, lycopene, α-carotene and β-carotene were found to be significantly lower in prostate cancer patients (*p* = 0.03, 0.008, 0.002 and 0.002, respectively). Plasma levels of elements such as iron, copper, calcium and sulphur were significantly higher (*p* < 0.0001, <0.0001, <0.0001 and *p* = 0.0003, respectively) while that of selenium was significantly lower (*p* = 0.002) in prostate cancer patients. Higher prostate cancer risk is significantly associated with plasma levels below the median of lycopene (OR: 2.24), α-carotene (OR: 2.13), β-carotene (OR: 1.97) and high levels above the median of iron (OR: 2.31), calcium (OR: 4.35) and sulphur (OR: 2.39). The results of this study suggest that the plasma nutriome could be a useful diagnostic of prostate cancer risk.

## 1. Introduction

Prostate cancer (PC) is one of the most common malignancies in men in developed countries. The aetiology of prostate cancer is largely unknown. Several risk factors such as ethnicity, family history and age have been shown to be associated with the increased prostate cancer risk [1,2,3].

It is also plausible that micronutrient deficiency or excess could cause DNA damage which is one of the hallmarks of cancer. Folate and vitamin B12 have a number of biologic roles such as the synthesis of purines and thymidylate for DNA synthesis and of methionine the precursor of s-adenosyl methionine (SAM) required for maintenance of DNA methylation [4,5]. In addition to its vital role in methylation, folate, in the form of 5,10-methylenetetrahydrofolate, may have a protective role in cancer development by promoting the synthesis of thymidylate from uracil, minimizing the misincorporation of uracil in DNA which could lead to the formation of DNA strand breaks [4,5,6].

Deficiency of folate, vitamin B6, and vitamin B12 are primary determinants of high blood homocysteine levels because they are essential for conversion of homocysteine to methionine or cysteine [7]. Vitamin E includes four tocopherols (α, β, γ, δ) and α-tocopherol is the most predominant and active form of vitamin E in humans thought to inhibit carcinogenesis through its antioxidant activity [8,9]. Retinoids have dominant effects on cell differentiation and proliferation and have been shown to be effective in the prevention of carcinogenesis in stem cells and cell lines [10,11]. Carotenoids and tocopherols have been suggested to be cancer preventive mainly because of their antioxidant properties, which may lead to a reduction in the extent of oxidative stress, lipid peroxidation and DNA damage, whereas retinol, along with pro-vitamin α-carotenoids, is involved in the control of cellular growth kinetics [12]. Oxidative stress and inflammation increase with age and is one of the factors related to prostate cancer risk [13]. Carotenoids may therefore be particularly relevant for preventing this disease [14,15]. There is some evidence that dietary carotenoids, retinol, and tocopherols may affect the development of prostate cancer and high concentrations of lycopene may be associated with a reduction in risk [16,17]. Higher circulating levels of α-cryptoxanthin, α-carotene, trans-α-carotene, and lutein and zeaxanthin was associated with lower prostate cancer risk in 118 non-Hispanic Caucasian men mainly from southeast Texas [18,19,20]. However, other reports did not find protective effects of lycopene and other carotenoids except for β-carotene against prostate cancer [21,22]. 

Many epidemiological and laboratory studies provided persuasive evidence that diet, genetic factors, and lifestyle were the major contributory factors of prostate cancer [23,24,25,26,27,28,29,30]. Some metal ions are essential for health; however, an overabundance or a deficiency of metal ions may lead to a cancerous state or progression of many types of cancer [31,32,33,34,35]. The anticancer activity of selenium has been attributed to its role in inducing apoptosis, inhibiting cellular proliferation, and being a key component of glutathione peroxidase, which protects cells from peroxide damage [36]. Selenium is a potential chemopreventive agent against prostate cancer. Serum selenium levels in prostate cancer cases were reported to be lower compared with controls [37,38]. Selenium is incorporated as the amino acid selenocysteine during translation into 25 selenoproteins many of which show a wide range of functions, including antioxidants and redox functions [39,40]. Iron an essential metal in human body can participate in many biological processes including the generation of highly reactive hydroxyl radicals (reactive oxygen species) that are thought to induce cancer [41]. Both a deficiency and excess of iron in blood is associated with increased DNA damage [42]. The fact that Zn is required as a cofactor in DNA replication and repair, is suggestive that deficiency in this micronutrient may induce important chromosomal mutations that increase cancer risk. It has been shown that Zn deficiency causes DNA oxidation, DNA breaks, and chromosome damage [43,44]. In the prostate gland, its concentration is 10 times higher than other organs; however, Zn is found to be decreased severalfold in cancerous tissue [45].

Emerging evidence suggests that various micronutrients, minerals and trace elements play an important role in cancer prevention. The present study was designed to test the hypothesis that the plasma levels profile of different micronutrients and trace elements in prostate cancer patients is substantially different from that of healthy controls.

## 2. Materials and Methods

### 2.1. Study Population

This hospital-based case–control study was conducted by a collaborative team from Royal Adelaide Hospital and CSIRO Human Nutrition between 2004 and 2006. The study design was approved by the Royal Adelaide Hospital and CSIRO Ethics Committees. All subjects gave written informed consent for participation.

All cases were untreated male Caucasian patients (*n* = 116) with histologically confirmed prostate cancer. The indication for prostate biopsy was a suspicious finding on digital rectal examination (DRE) and/or elevated serum levels of prostate-specific antigen (PSA; 0.08–45). The aggressiveness of the tumour in the prostate patients was defined by the Gleason score [46] and it varied between 6 and 9. All patients who were part of this study were classified as requiring radiotherapy for cancer control. Age-matched controls (*n* = 132) were male individuals who at that time were without any sign of cancer including prostate cancer, had a normal plasma PSA concentration and were not taking any medication for the treatment of life-threatening diseases. Cases and controls in this study were age matched. We did our best to match smoking status between groups but were limited by the difficulty in recruiting smokers in the control group. Table 1 describes the characteristics of cases and controls. 

A 20 mL blood sample was collected according to the standardized protocol. The blood samples were kept on ice, protected from light before being transferred to the laboratory for further analysis. 

### 2.2. Laboratory Assays

Plasma concentrations of homocysteine, vitamin B12 and folate were measured at the Institute of Medical and Veterinary Medicine (IMVS) using standard methods [47]. Plasma concentrations of carotenoids, retinol and α-tocopherol were measured by using HPLC within CSIRO Human Nutrition [48]. Plasma concentrations of various trace elements (minerals) such as selenium, zinc, iron, copper, calcium, magnesium, sodium, potassium, phosphorous and sulphur were analysed at CSIRO Plant Industry using inductively coupled plasma atomic emission spectroscopy (ICP-AES). All samples were first digested using nitric acid and hydrogen peroxide to ensure good recovery of all elements. Duplicate analyses were carried out for each sample. Both cases and controls were coded and analysed in the same batch to avoid inter-assay variation. Detailed description of the procedures is provided in the File S1. Immunoassay was used to detect total PSA in cases and controls.

### 2.3. Statistical Analysis

All data for each nutrient and other parameters were analysed for Gaussian distribution to determine whether to use parametric or non-parametric tests. Wilcoxson matched pairs test was used to test parametric data and Mann–Whitney U test for non-parametric data. Differences between groups were also compared using binary parameters (e.g., above/below median) with the chi-square test. We calculated odds ratios after classifying subjects based on whether their plasma micronutrient status was less than the median of the controls or ≥ than median of controls. Odds ratios were only calculated for those micronutrients that were significantly different in concentration between controls and prostate cancer cases. The results were also analysed with respect to combined selenium and lycopene concentration by comparing number of subjects with plasma concentrations >120 μmol and 0.25 μg/mL of controls Vs ≤ 120 μmol and 0.25 μg/mL of controls, respectively. All *p* values < 0.05 were considered statistically significant. All statistical analyses were performed using Graph Pad Prism 9.0 (Graphpad Inc., San Jose, CA, USA).

## 3. Results

Baseline characteristics of cases and controls are shown in Table 1. Cases and controls were closely matched on age. Mean age at study entry was 71.24 years for cases and 69.07 years for controls (*p* = 0.88). Total plasma PSA was 3.9-fold greater in cases relative to controls (*p* = 0.0001) and Gleason score ranged from 6 to 9 in prostate cancer cases. The number of current smokers and ex-smokers in cases (7.6% and 50.8%, respectively) was greater than in controls (2.3% and 29.5%), respectively.

**Figure 1:**
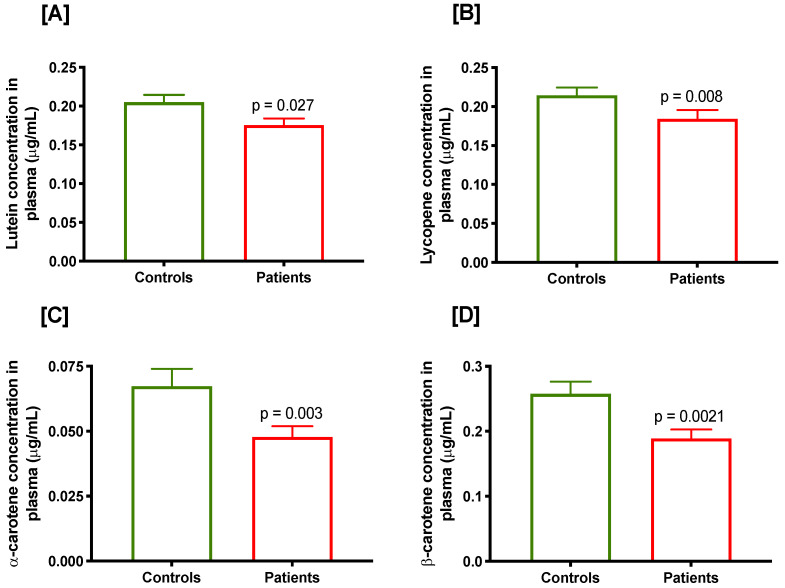
Plasma concentration of various carotenoids in controls and prostate cancer cases, (**A**) lutein, (**B**) lycopene, (**C**) α-carotene and (**D**) β-carotene.

Mean plasma concentrations of micronutrients in prostate cancer cases and controls are presented in Table 2. Plasma folate and B12 were slightly lower while homocysteine level (a biomarker of folate and/or B12 deficiency) was slightly higher in prostate cancer cases compared with controls but difference was not statistically significant (*p* ≥ 0.2). Lutein (*p* = 0.027; Figure 1A), lycopene (*p* = 0.008; Figure 1B), α-carotene (*p* = 0.003; Figure 1C) and β-carotene (*p* = 0.002; Figure 1D) were significantly lower in prostate cancer cases compared with the controls.

**Figure 2:**
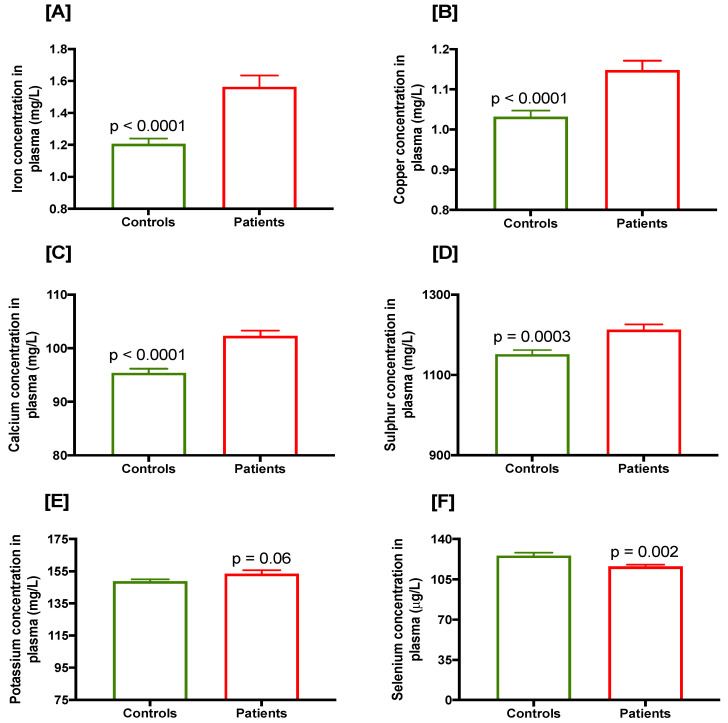
Plasma concentration of various trace elements in controls and prostate cancer cases, (**A**) iron, (**B**) copper, (**C**) calcium, (**D**) sulphur, (**E**) potassium and (**F**) selenium.

Mean plasma concentration of various elements in prostate cancer cases and controls are presented in Table 3. Mean plasma concentrations of iron, copper, calcium (Figure 2A–C) and sulphur (Figure 2D) were significantly higher in prostate cancer cases compared with controls (all *p* < 0.0001 except for sulphur which was *p* = 0.0003). Plasma concentrations of magnesium, sodium, phosphorous and potassium (*p* = 0.06; Figure 2E) were slightly but insignificantly higher in prostate cancer cases compared with controls. However, mean plasma selenium concentration in prostate cancer cases was significantly lower compared with controls (*p* = 0.002; Figure 2F).

Table 4 shows the associations of plasma micronutrients and elements that were significantly higher or lower in cases and their corresponding odds ratio (OR) for prostate cancer. Prostate cancer risk was significantly higher (OR: 2.24; 95% CI: 1.29–3.88; *p* = 0.006) if lycopene concentration was below the median value found in controls. Furthermore, prostate cancer risk was significantly higher (OR: 2.13; 95% CI: 1.23–3.69; *p* = 0.009) if α-carotene concentration was below the median value found in controls. Similarly, prostate cancer risk was increased almost two-fold if β-carotene concentration was below the median value of controls (OR: 1.97; 95% CI: 1.41–3.40; *p* = 0.01). However, if plasma iron concentration was above the median concentration of controls, prostate cancer risk increased by more than two-fold (OR: 2.31; 95% CI: 1.29–4.16; *p* = 0.006). Similarly, prostate cancer risk more than doubled (OR: 2.39; 95% CI: 1.33–4.27; *p* = 0.004), if the plasma sulphur concentration was more than the median concentration of controls. Prostate cancer risk was 4.35 times more likely if the median plasma calcium concentration was higher than the median for control (OR: 4.35; 95% CI: 2.24–8.44; *p* < 0.0001). We did not find a significant OR for prostate cancer risk relative to plasma copper, selenium or lutein when using the median of controls for stratification of data. When nutrient-nutrient interactions were studied, we found that prostate cancer risk was increased nearly threefold (OR: 2.97; 95% CI: 1.19–7.41; *p* = 0.019) if plasma level of selenium and lycopene are ≤120 μmol and ≤ 0.25 μg/L, respectively.

Plasma concentration of selenium was positively correlated with lutein (r = 0.152; *p* = 0.11), α-tocopherol (r = 0.173; *p* = 0.07) and lycopene (r = 0.134; *p* 0.16) in controls. However, we were unable to find this correlation in patients. Plasma selenium and zinc were significantly positively correlated (r = 0.273; *p* = 0.006) in patients only. Similarly, α-tocopherol is positively associated with lycopene (r = 0.161; *p* = 0.09), α-carotene (r = 0.205; *p* = 0.03) and β-carotene (r = 0.280; *p* = 0.003), lutein (r = 0.256; *p* = 0.006), and lutein with lycopene (r = 0.335; *p* = 0.000) in controls only. 

We also calculated a nutrient risk index (NRI) for prostate cancer by determining for each case and control whether their plasma concentrations of lycopene, α-carotene and β-carotene was below the median of controls, and whether they were above the median for iron, sulphur and calcium. A score was given for each instance where plasma nutrient concentration was on the risk side so that the worst score was 6 and the best 0. NRI data were then used to calculate the OR of being a prostate cancer case. As shown in Table 5, the OR was highest for those with an NRI of 6 and likelihood ratio increased steadily from 1.04 to 4.08 as the NRI increased progressively from 1 to 6.

## 4. Discussion

This is the first preliminary study to evaluate the plasma concentrations of micronutrients and trace elements with respect to prostate cancer in the South Australian population. 

Folate and vitamin B12 are essential for nucleotide synthesis, DNA methylation, repair, and replication [49]. Folate is an essential water-soluble vitamin and key cofactor in one-carbon metabolism that can regulate many other pathways such as cell growth, differentiation, DNA-repair, apoptosis, and carcinogenesis prevention [50]. Dietary folate deficiency has been associated with the increase of some cancers while excessive supplementation may also be linked with increased cancer risk [51,52]. Vitamin B12 influences the bioavailability of folate in cells in its role as co-factor for methionine synthesis and is essential for maintaining myelination of neurons as well as haematopoiesis. Vitamin B12 deficiency can lead to irreversible neurological damage and/or megaloblastic anaemia [53,54,55]. The results from the ProtecT case–control study found no association of folate with prostate cancer risk. The results from this same study strongly influenced the outcome of a meta-analysis of prostate cancer studies, which would otherwise have shown a clear positive association of circulating folate with increased prostate cancer risk [56,57]. It has also been shown that high circulating concentrations of vitamin B12 may be associated with increased risk of prostate cancer [56]. However, in our cohort, plasma folate and vitamin B12 concentrations were found to be marginally lower (*p* = 0.2 and *p* = 0.64, respectively) in PC patients compared with controls. The association between serum folate and cancer is subject to effect modification by age, smoking, genetic background and the time, duration and dose of folic acid supplementation. Furthermore, it has been shown that vitamin B12 and B6 influence serum concentrations of free folic acid [58]. Therefore, the association between folate and diseases is subject to confounding by several risk factors that are known to influence serum concentrations of free folic acid and the disease outcome. However, a recent pooled analysis from observational data indicates a weak positive association between circulating folate and vitamin B12 and risk of PC [59]. We found no associations of homocysteine with prostate cancer risk and is in agreement with previously published reports [56,60,61].

Carotenoids are pigments found in nearly all coloured fruits and green leafy vegetables and their consumption has been associated with various health benefits, including a reduced risk for some cancers [62]. Carotenoids may guard against certain types of cancer by limiting the abnormal growth of cells and/or by enhancing gap-junctional communication [63]. Lycopene, α-carotene, β-carotene, and lutein represent the major carotenoids in the human diet. Lycopene is found in relatively high concentrations within the prostate gland [64] and some epidemiological studies have found an inverse association between lycopene intake and prostate cancer [65,66]. In our cohort, plasma lycopene concentration was found to be significantly low in prostate cancer patients relative to controls. It is plausible that lycopene might reduce cancer risk by acting as an antioxidant, inhibiting cell proliferation or inducing apoptosis [12,16,66,67]. However, a recently published report from pooled analysis did not find circulatory lycopene to be associated with prostate cancer risk [68]. It has been reported that although circulating α-carotene and β-carotene were positively (weakly) associated with prostate cancer risk in the unadjusted analyses, but after adjustment for anthropometric and lifestyle factors, these associations become null [68]. In the present study, plasma concentration of carotenoids such as lutein, lycopene, α-carotene and β-carotene were found to be significantly lower in prostate cancer patients. However, there was no statistically significant association between plasma carotenoids concentration with serum PSA either in control or prostate cancer patients. Similarly, α-tocopherol was found to be significantly but inversely associated with prostate cancer risk, and this association appeared to be restricted only to advanced and aggressive prostate cancer [68]. In the present case–control study, we did not find that α-tocopherol was associated with a protective role against prostate cancer.

Retinol is found in dairy and flesh foods. Plasma retinol levels may indicate dietary intake of retinol [69]. In a recently published meta-analysis, high retinol concentration was found to be significantly associated with increased prostate cancer risk [68]; however, we did not find any indication of increased risk for prostate cancer with plasma retinol.

A growing body of evidence has suggested that trace elements play a vital role in many biological processes either by activating or inhibiting enzymes, by competing with other elements and metallo-proteins for binding sites, by influencing cell membrane permeability or by other mechanisms [70]. It is, therefore, reasonable to assume that these trace elements may act directly or indirectly on the carcinogenic process [71]. Selenium is a naturally occurring element and has both nutritional and toxicological effects that have received substantial attention in medicine and biology [72]. Trace amounts of selenium are necessary for cellular function and for the synthesis of several selenoproteins having antioxidant properties such as glutathione peroxidases (GPX), thioredoxin reductases and selenophosphate synthetase 2 [73]. A recent analysis from fifteen prospective studies showed some protective effects of selenium in patients with aggressive form of prostate cancer [74]. Another recent study only found weak evidence of a positive effect of selenium on advanced prostate cancer [75]. In our cohort, we found significantly higher blood selenium concentration (*p* = 0.002) in controls compared with PC patients. Similar observation was repeated in a recent meta-analysis from various observational studies [38]. This may be attributed to selenium’s superior scavenging of reactive nitrogen oxide species, and also to its capacity to decrease inflammation [38,76]. In addition, selenium has also been shown to inhibit cell proliferation and decreases cell cycle progression through the reduction of cyclin in prostate cancer cell lines [38]. Iron is required for cell proliferation and earlier studies have hypothesised that iron can increase growth of neoplastic cells through inflammation by suppressing host defence mechanisms [77]. Previous studies have found an association between high iron levels and increased prostate cancer risk [78,79] whereas a previous study did not show any significant difference in iron levels in prostate cancer subjects compared with controls [80]. In the present study, blood iron level is significantly higher in PC patients relative to control. 

The 2014 World Cancer Research Fund report on prostate cancer indicated that dairy products (rich source of calcium) may be associated with a limited but suggestive increased risk for prostate cancer incidence [81]. This conclusion was further substantiated by a meta-analysis which suggested that high intake of dairy products and calcium are associated with increased risk (RR: 1.09; 95% CI: 1.02–1.17) of prostate cancer [82]. It has been suggested that an increased circulating concentration of IGF-1 might be associated with an increased calcium intake and prostate cancer risk [83,84]. High calcium levels in blood have been found to be associated with an increased risk of fatal prostate cancer [85]. In the present study, we found significantly higher level of plasma calcium in prostate cancer patients relative to controls. However, another recent study reported null results with regard to calcium [86]. It is possible that higher calcium levels in blood may be due to higher intake of calcium rich food or altered absorbance or uptake by the cells. It has also been hypothesized that dietary calcium may indirectly increase PC risk and promote tumour growth by reducing blood levels of the biologically active form of vitamin D (1,25-dihydroxy cholecalciferol) that helps in inhibiting proliferation of prostate cells [87]. 

Copper is an essential trace element required for the activity of a large number of enzymes (ceruloplasmin, Cu/Zn superoxide dismutase, hephaestin, etc.) and structural proteins [88], but its high concentration could induce oxidative stress and growth proliferation predominantly due to its ability to change between Cu(I) and Cu(II) leading to generation of ROS [89,90]. It has been shown previously that circulating copper levels are elevated in patients with prostate cancer [91,92,93,94]. It has also been suggested that copper may activate RAS/MAP kinase pathway and induce angiogenesis and could potentially promote growth of prostate cancer by improving blood supply [95]. In the present study, we found significantly higher plasma concentration of copper in prostate cancer patients relative to controls. It is not yet clear why prostate cancer patients have such a high concentration of copper. 

Sulphur is an essential element for cellular functions and is required for synthesis of essential amino acids such as methionine and cysteine. It has been shown that sulphur concentration in blood is significantly higher in bladder cancer patients than controls [96]. However, we did not find any other report that studied the blood sulphur concentration either in prostate cancer or other cancers. In this cohort, we found significantly higher concentration of sulphur in PC patients relative to controls. It is possible that this is simply a co-incidence of higher dietary intake of proteins rich in sulphur amino acids (e.g., eggs, red meat, poultry and fish) or perhaps altered absorption of sulphur by the cells. Tumour-derived hydrogen sulphide [97] and allosteric formation of disulphide bonds in cancer-related proteins [97] might be the possible cancer promoting mechanisms; however, it is still unclear how prevalent is the formation of these compounds when concentration of this trace element (sulphur) is high in blood. If the high plasma sulphur can be explained by high methionine concentration, it is plausible that this could lead to an increase in the supply of S-adenosylmethionine (SAMe), the common methyl donor. Furthermore, high methionine and high SAMe are required by prostate cancer cells for their growth and survival [98,99].

We did not find any significant differences in the blood concentration of other elements such as zinc, sodium, potassium, magnesium and phosphorus in this cohort.

## 5. Conclusions

Well-designed future prospective studies should address reasons of alterations in serum or plasma concentrations of micronutrients in patients with prostate cancer and clarify their relationship with various stages of prostate cancer. The results of this study highlight that (i) increased plasma calcium, sulphur and iron, and (ii) decreased selenium, lycopene, α-carotene and α-carotene are all related with increased PC risk. These micronutrients are also indicators of increased intake of a Western dietary pattern rich in dairy and flesh food, and deficient in plant foods. Therefore, it would be interesting and important to verify whether these biomarkers consistently reflect Western dietary pattern and are also predictors of prostate cancer risk in other population, within Australia and other countries. Overall, the results of our study support the proposal that plasma micronutrient profiles can serve as biomarkers of prostate cancer risk.

## Figures and Tables

**Table 1:** Comparison of prostate cases and controls by selected demographic and clinical variables.

Characteristics	Cases	Controls	*p* Value
Age (years; Mean ± SD)	71.24 ± 7.18	69.07 ± 7.99	0.88
Total plasma PSA (ng/mL; mean ± SD)	9.5 ± 8.5	2.4 ± 2.45	0.0001 *
Gleason Score	6–9	-	-
Smoking Status			
Current Smokers	9	3	0.0001 *
Ex-Smokers	60	39	
Non-Smokers	25	54	
Undeclared	24	36	

* Chi-square test.

**Table 2:** Mean (±S.E.) plasma concentrations of various micronutrients in prostate cancer patients and controls.

Micronutrients/ Trace Elements	Controls	Prostate Cancer	*p* Value
Homocysteine (μmol/L)	10.27 ± 0.28 (5.5–23.4)	12.21 ± 1.009 (4.6–94.0)	0.25
B_12_ (pmol/L)	284.5 ± 11.4 (121–862)	276.8 ± 15.97 (44–1450)	0.24
Folate (nmol/L)	19.26 ± 0.78 (5.4–37.7)	18.22 ± 0.96 (2.9–45)	0.2
Lutein (μg/mL)	0.205 ± 0.009 (0.06–0.64)	0.176 ± 0.009 (0.029–0.553)	0.027 *
Retinol (μg/mL)	0.678 ± 0.015 (0.38–1.32)	0.654 ± 0.015 (0.414–1.086)	0.29
α-Tocopherol (μg/mL)	13.17 ± 0.36 (6.35–29.4)	13.31 ± 0.42 (7.07–37.48)	0.63
Lycopene (μg/mL)	0.215 ± 0.009 (0.03–0.58)	0.184 ± 0.011 (0/013–0.655)	0.008 *
α-Carotene (μg/mL)	0.067 ± 0.006 (0.01–0.61)	0.048 ± 0.004 (0.002–0.273)	0.003 *
β-Carotene (μg/mL)	0.258 ± 0.019 (0.03–1.36)	0.189 ± 0.014 (0/017–0.704)	0.002 *

Values given in brackets represent the range. * Chi-square test.

**Table 3:** Mean (±S.E.) plasma concentrations of various trace elements in prostate cancer patients and controls.

Trace Elements	Controls	Prostate Cancer	*p* Value
Selenium (μg/L)	125.6 ± 2.56 (79.17–238.1)	116.1 ± 1.59 (71.83–157.6)	0.002 *
Zinc (μg/mL)	0.78 ± 0.01 (0.52–1.04)	0.77 ± 0.1 (0.52–1.24)	0.23
Iron (mg/L)	1.207 ± 0.03 (0.5–2.3)	1.564 ± 0.07 (0.52–4.7)	<0.0001 *
Copper (mg/L)	1.032 ± 0.015 (0.6–1.5)	1.148 ± 0.023 (0.58–2.0)	<0.0001 *
Calcium (mg/L)	95.4 ± 0.79 (79–119)	102.3 ± 1.006 (77–128)	<0.0001 *
Magnesium (mg/L)	21.35 ± 0.208 (17–28)	21.78 ± 0.261 (15–27)	0.19
Sodium (mg/L)	3338 ± 22.89 (2835–4095)	3395 ± 27.41 (2600–4000)	0.19
Potassium (mg/L)	148.9 ± 1.18 (114–182)	153.5 ± 2.22 (100–222)	0.06
Phosphorus (mg/L)	124.6 ± 1.81 (87–176)	128.2 ± 2.15 (81–189)	0.19
Sulphur (mg/L)	1152 ± 10.19 (960–1403)	1213 ± 13.18 (870–1560)	0.0003 *

Values given in brackets represent the range. * Chi-square test.

**Table 4:** Odds ratio in relation to plasma concentration of various micronutrients and trace elements and prostate cancer risk using median values for control as cut off.

	Micronutrient/ Trace Element	Odds Ratio (95% CI)	*p* Value
**Micronutrients which Tend to be Higher in Controls**	**Lutein**		
	≥Median (0.18 μg/mL)	1 (Ref)	
	<Median (0.18 μg/mL)	1.35 (0.79–2.31)	0.28
	**Lycopene ^#^**		
	≥Median (0.205 μg/mL)	1 (Ref)	
	<Median (0.205 μg/mL)	2.24 (1.29–3.88)	0.006 *
	**α-Carotene ^#^**		
	≥Median (0.05 μg/mL)	1 (Ref)	
	<Median (0.05 μg/mL)	2.13 (1.23–3.69)	0.009 *
	**β-Carotene ^#^**	1 (Ref)	
	≥Median (0.21 μg/mL)	1 (Ref)	
	<Median (0.21 μg/mL)	1.97 (1.41–3.40)	0.01 *
	**Selenium**		
	≥Median (118.5 μmol/L)	1 (Ref)	
	<Median (118.5 μmol/L)	1.36 (0.79–2.33)	0.28
**Micronutrients which Tend to be Lower in Controls**	**Iron ^#^**		
	<Median (1.2 mg/L)	1 (Ref)	
	≥Median (1.2 mg/L)	2.31 (1.29–4.16)	0.006 *
	**Copper**		
	<Median (1 mg/L)	1 (Ref)	
	≥Median (1 mg/L)	0.94 (0.5–1.77)	0.87
	**Calcium ^#^**		
	<Median (94 mg/L)	1 (Ref)	
	≥Median (94 mg/L)	4.35 (2.24–8.44)	<0.0001 *
	**Sulphur ^#^**		
	<Median (1150 mg/L)	1 (Ref)	
	≥Median (1150 mg/L)	2.39 (1.33–4.27)	0.004 *
**Selenium/Lycopene ^#^**			
>120 Selenium/>0.25 Lycopene		1 (Ref)	
≤120 Selenium/≤0.25 Lycopene		2.97 (1.19–7.41)	0.02 *

^#^: micronutrients and trace elements significantly different in two groups. * Chi-square test.

**Table 5:** Summary data describing the odds of being a prostate cancer case with increasing Micronutrient Risk Index.

	Micronutrient Risk Index					
	≥1	≥2	≥3	≥4	≥5	6
*p* Value	0.13	0.005	0.0003	< 0.0001	< 0.0001	0.001
Odds Ratio (95% CI)	3.23 (0.64–16.41)	4.03 (1.42–11.4)	3.37 (1.72–6.58)	3.14 (1.75–5.62)	3.89 (2.09–7.24)	4.88 (1.76–13.54)
RR (95% CI)	2.07 (0.62–6.94)	2.37 (1.09–5.22)	1.98 (1.28–3.05)	1.79 (1.3–2.46)	1.83 (1.41–2.38)	1.75 (1.36–2.24)
Sensitivity	0.98	0.95	0.84	0.69	0.51	0.21
Specificity	0.06	0.17	0.38	0.58	0.78	0.95
PPV	0.52	0.54	0.59	0.63	0.71	0.81
NPV	0.75	0.77	0.7	0.65	0.61	0.54
Likelihood Ratio	1.04	1.15	1.38	1.66	2.4	4.08

RR: Relative risk; PPV: Positive predictive value; NPV: Negative predictive value.

## Data Availability

Data will be uploaded to a publicly available repository upon acceptance of the manuscript.

## Supplementary Materials

The following supporting information can be downloaded at: https://www.mdpi.com/article/10.3390/cancers15010077/s1, File S1: Analysis of carotenoids and minerals.
