# Supplementary material for: Plasma Micronutrient Profile of Prostate Cancer Cases Is Altered Relative to Healthy Controls—Results of a Pilot Study in South Australia
Source: Cancers (Basel). 2022 Dec 23;15(1):77. doi: 10.3390/cancers15010077 (PMC9817984; doi:10.3390/cancers15010077)
Supplement: Supplementary file 1 [file cancers-15-00077-s001.zip › cancers-2087885-supplementary.pdf]

Supplementary materials

Title: Analysis of carotenoids and minerals

Analysis of Carotenoids

Blood samples were collected in tubes containing EDTA as an anticoagulant. The plasma was separated by low-speed centrifugation at 600 x g for 10 min at 5°C (GS-6R centrifuge), frozen immediately in liquid nitrogen, and then stored at -80°C until analysis. Plasma extractions and HPLC chromatography were performed according to the methods of Yang and Lee [1], Khachik et al [2] and Record et al [3].

Only a few samples were processed at a time to minimize the exposure to laboratory conditions. The lighting was minimal throughout sample preparation, and amber vials were used for storage of the final extract. The internal standard (α-tocopherol acetate) was added to samples (200 µL) with 200 µL ethanol. Vitamins and carotenoids were extracted with hexane, and the extract was evaporated to dryness under nitrogen. Extracts were then stored at -20°C.

A Shimadzu LC 10 HPLC (Shimadzu, Kyoto, Japan) fitted with a refrigerated autosampler and an SPD-M10Avp photodiode array detector with a class LC10 chromatography workstation was used for analysis of the prepared samples. Isocratic separations of the fat-soluble vitamins and carotenoids were carried out on a Rainin (Walnut CK, CA, USA) (4´6 mm i.d.250 mm length) C18 (5m spherical particles) reverse-phase column. The mobile phase was a mixture of (%, v/v): acetonitrile 55%, methanol 22%, hexane 11.5%, dichloromethane 11.5%; the flow rate was 1.0 ml/min. Ammonium acetate (0.01 %, w/v) was added to the mobile phase for stabilisation of the carotenoids. α-carotene, β-carotene, lycopene, lutein, retinol, α-tocopherol and α-tocopheryl acetate standards were all obtained from Sigma-Aldrich Chemicals. Solvents, hexane, methanol, acetonitrile, ethanol and dichloromethane were all of analytical HPLC grade. A standard reference material (National Institute of Standards and Technology product 968b) was analysed with each batch of samples. The reference material with certified concentrations of retinol, α-tocopherol, lutein and β-carotene was used to monitor variation in analyses. All samples from a volunteer were extracted in duplicate and analysed in one run on the HPLC instrument to minimize the effect of day-to-day variation. CVs for each of the carotenoids were as follows: lutein, 2.5%; retinol, 2.5%; α-tocopherol, 2.9%; lycopene, 2.7%; α-carotene, 2.9%; and β-carotene, 2.7%.

References

1. Yang CS, Lee M-J. Methodology of plasma retinol, tocopherol and carotenoid assays in cancer prevention studies. J Nutr Growth Cancer 1987;4:19–27.
2. Khachik F, Beecher GR, Goli MB, Lusby WR, Smith JC Jr. Separation and identification of carotenoids and their oxidation products in the extracts of human plasma. Anal Chem 1992;64:2111–22.
3. Record IR, Dreosti IE, McInerney JK. Changes in plasma antioxidant status following consumption of diets high or low in fruit and vegetables or following dietary supplementation with an antioxidant mixture. Br J Nutr. 2001 Apr;85(4):459-64.

Analysis of Minerals

Plasma concentrations of the elements zinc, iron, copper, calcium, magnesium, sodium, potassium, phosphorous and sulphur were analysed at CSIRO Plant Industry using inductively coupled plasma atomic emission spectroscopy (ICP-AES) (Varian Liberty Series II). All samples were first digested using nitric acid and hydrogen peroxide to ensure good recovery of all elements. Se concentrations in plasma were determined by inductively coupled plasma mass spectrometry (ICPMS) (Agilent Technologies 7500c, Japan), following digestion with nitric acid and hydrochloric acid as previously described [1].

Accuracy of measurement of trace elements and heavy metals was assured by analysis of certified reference material using Seronorm™ Trace Elements Serum (Lot JL4409). There was < 5% variation from the expected value of the Seronorm reference material.

Duplicate analyses were carried out for each sample. The CVs for repeat sample analysis of each element were as follows: Ca 1.8%; Cu 3.3%; Fe 2.2%; K 5.1%; Mg 2.6%; Na 1.4%; P 1.6%; S 1.3%; Zn 4.2%; Se 1.5%.

References:

1. Wu J, Salisbury C, Graham R, Lyons G, Fenech M. Increased consumption of wheat biofortified with selenium does not modify biomarkers of cancer risk, oxidative stress, or immune function in healthy Australian males. Environ Mol Mutagen. 2009 Jul;50(6):489-501. doi: 10.1002/em.20490. PMID: 19365873.
